# Variable Mutation Rates as an Adaptive Strategy in Replicator Populations

**DOI:** 10.1371/journal.pone.0011186

**Published:** 2010-06-17

**Authors:** Michael Stich, Susanna C. Manrubia, Ester Lázaro

**Affiliations:** Department of Molecular Evolution, Centro de Astrobiología (CSIC-INTA), Torrejón de Ardoz, Madrid, Spain; University of Zurich, Switzerland

## Abstract

For evolving populations of replicators, there is much evidence that the effect of mutations on fitness depends on the degree of adaptation to the selective pressures at play. In optimized populations, most mutations have deleterious effects, such that low mutation rates are favoured. In contrast to this, in populations thriving in changing environments a larger fraction of mutations have beneficial effects, providing the diversity necessary to adapt to new conditions. What is more, non-adapted populations occasionally benefit from an increase in the mutation rate. Therefore, there is no optimal universal value of the mutation rate and species attempt to adjust it to their momentary adaptive needs. In this work we have used stationary populations of RNA molecules evolving *in silico* to investigate the relationship between the degree of adaptation of an optimized population and the value of the mutation rate promoting maximal adaptation in a short time to a new selective pressure. Our results show that this value can significantly differ from the optimal value at mutation-selection equilibrium, being strongly influenced by the structure of the population when the adaptive process begins. In the short-term, highly optimized populations containing little variability respond better to environmental changes upon an increase of the mutation rate, whereas populations with a lower degree of optimization but higher variability benefit from reducing the mutation rate to adapt rapidly. These findings show a good agreement with the behaviour exhibited by actual organisms that replicate their genomes under broadly different mutation rates.

## Introduction

Mutations constitute the main source of genetic diversity in asexual populations. Although most of them have deleterious effects on fitness [1]–[3], natural selection increases the representation of those having beneficial effects, which can become fixed in a population. This combined action of mutation and selection promotes the adaptation to environmental changes, and in the long term leads to the evolution of populations.

In the framework of the fitness landscape described by Wright [4], populations placed near the top of a fitness peak will experience less beneficial mutations than populations placed far from the adaptive optimum [2], [5]–[9]. This dependence of the mutation effects on the degree of adaptation of populations led to the theoretical prediction that mutation rates would be reduced in constant environments, in which the population has had enough time to adapt. Once the optimum has been attained, a homogeneous population of individuals with the optimal phenotype is the best adaptive solution, so the generation of further diversity is not necessary. Nevertheless, the actual situation is that environments never remain static. They continuously undergo changes that alter the fitness landscapes, displacing populations towards suboptimal fitness regions, where the amount of mutations with positive effects increases. These poorly-adapted populations could benefit from having higher than standard mutation rates.

The variation of mutation effects with fitness, together with the fact that error rates can be easily modified as a consequence of mutations producing genotypes with variable capacity to cause errors, suggest that mutation rates are a character subjected to the action of natural selection [10], [11]. Stable environments would favour low mutation rates (anti-mutator genotypes), constrained only by the costs of error-repair mechanisms [12], [13]. In contrast to this, environments subjected to frequent changes would select for increased mutation rates (mutator genotypes) that permit faster adaptation to the new conditions [14]–[16]. However, the optimization of the mutation rate is not only determined by its impact on adaptation but also by the consequences that the variation of this character has on fitness. High mutation rates can increase the number of deleterious mutations, whereas low mutation rates can have metabolic costs associated. The existence of these opposing forces causes that natural selection often fails to fully optimize this character [17]. The study of the evolution of mutation rates has been addressed theoretically [13], [18]–[21], and using digital organisms [17]. There are also many reported examples of natural and experimental bacterial populations with higher than standard mutation rates [22]–[26], showing that there are multiple situations in Nature in which being a mutator confers a selective advantage. Although mutator variants have also been isolated in the DNA phage T4, they have been rarely observed in the case of RNA viruses [27], [28]. A possible explanation is that RNA viruses replicate their genomes at the maximum error rate compatible with the preservation of genetic information, and additional increases would lead to fitness losses that could cause the extinction of the population [29], [30]. Despite this almost absence of mutators in RNA viruses, it has been observed that low fitness clones of an RNA bacteriophage increase their replicative ability when infections take place in the presence of a mutagen, a clear example of the adaptive advantages arising from an increase in the mutation rate in low fitness populations [31].

Anti-mutator mutants, with lower than normal mutation rates, have been observed in bacteria [32], in the phage T4 [33], and in RNA viruses evolving in the presence of mutagens [34]. In the latter case, the anti-mutator phenotype can be produced by single changes in the viral polymerase, without requiring the expression of corrector activities. These observations suggest that RNA viruses could easily evolve to lower mutation rates. If they do not, it could be due to the major adaptive advantages provided by high mutation rates. The finding that high-fidelity genotypes of an RNA virus have lost some of their adaptive properties in mice constitutes a strong support of this hypothesis [35], [36]. Other studies, however, point to the existence of a trade-off between rapid replication and fidelity to explain the high mutation rates of RNA viruses [37].

Asexual populations of replicators, such as RNA molecules evolving *in silico*, with selection acting on their folded conformation, constitute a simple system to study how the variation of the mutation rates influences adaptation. After a sufficiently long time, these virtual populations reach a stationary state characterized by mutation-selection equilibrium and a quasispecies structure [38]. Populations of RNA molecules have been very successfully used as a computational model for the study of evolutionary processes [39]–[41]. The influence of the mutation rate on the degree of adaptation attained at the stationary state, and on the genotypic and phenotypic diversity of the population are questions that have been addressed previously with this model [9], [42]. In this work we focus on the adaptability of populations of RNA molecules that reached the stationary state at different error rates, and that are affected by a sudden environmental change. To this end, we determine those mutation rates promoting maximal adaptation after a short number of generations. In practice, our population evolves under selection for folding into a given secondary structure until mutation-selection equilibrium is reached. At that point, it is confronted with a new selective pressure, represented by a new target structure, towards which it evolves under a second mutation rate. Our results show that, before reaching the new equilibrium, and especially at the early stages of the adaptive process, there is no simple relationship between the momentary degree of adaptation and the new error rate at which a population evolves. There is also a strong influence of the mutation rate at which populations evolved towards the previous stationary state in their ability to adapt to new selective pressures. Our results are of relevance to understand the adaptive process in changing environments when variations of the mutation rate are allowed. Some actual examples of this situation are the *in vitro* evolution of structural or catalytic RNA molecules and proteins -where the experimenter can manipulate the extension of the genetic diversity generated- the selection of mutator variants of pathogenic bacteria in response to antibiotics, hampering the treatment of many diseases [43], [44], and also RNA viruses in which even very mild mutator or antimutator phenotypes can have important consequences in shaping not only virus evolution, but also pathogenesis, transmission, and emergence [28], [35], [36]. A deeper knowledge of how mutation rates can affect fitness and adaptive ability can be of great importance to evaluate the effectiveness and long term consequences of therapies, especially those based on the increase of the mutation rate through the use of mutagens [45].

## Methods

### Evolutionary algorithm

The system used in our simulations consists of a population of *N* = 1000 RNA sequences, each of length *l* = 50 nucleotides. At the beginning of the simulation, each molecule of the population is initialized with a random sequence. Every time that a sequence replicates, each of its nucleotides has a probability (defined by the mutation rate *μ*) to be replaced by another nucleotide, randomly chosen among the four possibilities. We define a target secondary structure which is endowed with the highest replication rate. After each replication event, the molecules are folded into secondary structures with help of the Vienna package, version 1.5 [46] and the base pair distance *d_i_* between each molecule *i* in the population and the target is calculated. The base pair distance is defined as the number of base pairs that have to be opened and closed to transform a given structure into the target structure. The probability *p*(*d_i_*) that a molecule *i* in the population replicates depends on the distance *d_i_* according to the following equation:
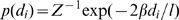
(1)where *Z* is the overall normalization factor *Z* = 
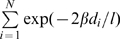
. The parameter *β* denotes the selection pressure and takes the value *β* = 1 for all the simulations carried out in this study; *l* is the length of the molecule. Generations are non-overlapping and the offspring generation is selected following a Wright-Fisher sampling at each time step.

At any time point, the population can be characterized by two main quantities: the fraction of molecules correctly folded (those with *d_i_* = 0) and the average distance of the population to the target structure. Because of the dependence of the probability of replication of each molecule on its *d_i_* value, we chose the average distance to the target as an estimator of the degree of adaptation of a population (the higher the distance the lower the degree of adaptation).

### Stationary, non-adapted, and adapting populations

**Figure 1:**
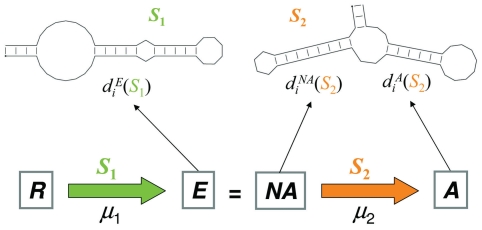
Schematic representation of the protocol undergone by the populations of RNA molecules. The simulation begins with a population of random sequences (*R*). It evolves towards the target structure *S*_1_ under a mutation rate *μ*_1_. After a number of generations that depends on the population size and on the mutation rate, mutation-selection equilibrium is attained. These are populations *E*, which after a sudden change in the target structure become non-adapted (populations *NA*) and are used as the initial condition for adaptation to the new environment. Then the mutation rate changes to *μ*_2_ and the populations adapting to the new secondary structure (*A*) are analysed during 200 generations. The degree of adaptation is quantified through the average distance of the population to each of the targets. In this work we pay particular attention to the distribution of distance values of populations at equilibrium, *E*, to target structure *S*_1_, 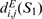, of non-adapted populations to target structure *S*_2_, 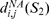, and to that of the adapting population *A* to target structure *S*_2_, 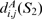. See main text for further details.

A general scheme showing the main evolutionary characteristics of the different populations used in our simulations is represented in Fig. 1.

The initial random populations *R* replicate with different mutation rates (*μ*_1_ = 0.001, 0.002, 0.005, 0.01, 0.02, and 0.05) during 8000 generations (*g*), a number large enough to reach a statistically stationary state, which is determined by the selective pressure represented by the target structure *S*_1_ (the hairpin structure shown in Fig 1). We performed *r* = 50 independent realizations of this process for each value of the mutation rate. The resulting equilibrated populations (populations *E*) experience a sudden change in the selective pressure implemented as a change of the target structure from *S*_1_ to *S*_2_ (the hammerhead represented in Fig. 1b). At this point, populations become non-adapted (populations *NA*), as they have not been optimized in the new environment defined by *S*_2_. We randomly choose one of the 50 non-adapted populations as the starting point of a new adaptive process towards *S*_2_. The population evolves during 200 generations under 6 different values of the mutation rate (*μ*_2_ = 0.001, 0.002, 0.005, 0.01, 0.02, and 0.05) giving rise to the adapting populations (populations *A*) shown in Fig. 1. We performed *r* = 1000 independent realizations for each value of the mutation rate. The average distance was determined for each adapting population at every generation, and averaged over the 1000 independent runs.

### Nomenclature

We focus our analyses in the three populations *E*, *NA*, and *A*. In the following, we will call *d_i,j_^E^*(*S*_1_) the set of base pair distances between each molecule *i* in populations *E* and the target structure *S*_1_ for each realization *j*. Likewise, we call *d_i,j_^NA^*(*S*_2_) the set of base pair distances between each of the molecules in populations *NA* and *S*_2_ for each of the realizations. It is important to remark that populations *E* and *NA* have the same composition. The only difference between them is the structure used to calculate the *d_i,j_* values, which in populations *E* has been the target of a completed optimization process whereas in populations *NA* has not. Analogously, we define *d_i,j_^A^*(*S*_2_) as the base pair distance between molecules in the adapting populations *A* and the target structure *S*_2_ for each realization *j*. Note that the latter set of distances is a time-dependent quantity, while *d_i,j_^E^*(*S*_1_) correspond to populations at the mutation-selection equilibrium, and are thus independent of time. Average values are calculated over the *N* = 1000 molecules in the population and over *r* independent realizations of the process. We first define the average distance to target for each realization *j* as, 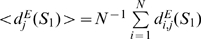. The average over the *r* independent realizations is 
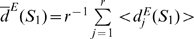
. As indicated, this is an important quantity used as a measure of the degree of adaptation of populations. Similar quantities are defined for non-adapted *NA* and adapting populations *A*. The number of realizations is *r* = 50 for *E* and *NA* populations and *r* = 1000 for *A* populations. For adapting populations *A* we calculate the standard deviation 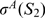 of the average distances 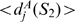 with respect to 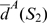 in order to measure the variability among realizations. The statistical significance of the differences between average distance values was determined with the Student's *t*-test. To get a deeper characterization of populations *E* and *NA*, we also determined several additional statistical parameters. We call 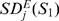 the standard deviation of the distances 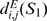 with respect to 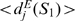. The average over standard deviations for the *r* realizations is 
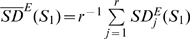
. The minimum and maximum distances in each set 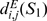 are called 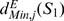 and 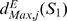, respectively. The averages over realizations are 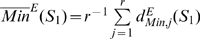 and 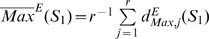. The skewness of the set 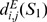 is called 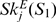, and the average over realizations is 
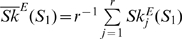
. Analogous quantities are defined for *NA* populations. All statistical calculations were performed with the program Mathematica 5.0 (Wolfram Research).

## Results

### Distributions of distance values in equilibrated and non-adapted populations

The structure of the populations used in this study can be described through the distribution of distance values (see Methods), which depends on the value of the mutation rate, the selective pressure dominant, and the time of evolution. The statistical parameters characterizing the distribution of distances to targets *S*_1_ and *S*_2_ in equilibrated and non-adapted populations are reported in Tables 1 and 2, respectively. Given the population structure at the stationary state, 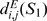, and the new selective pressure, *S*_2_, the set 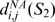 is completely determined, illustrating how the composition of populations at equilibrium influences its subsequent evolution.

**Table 1:** Statistical parameters describing the distribution of 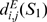 values (see Methods and Fig. 1).

*μ*_1_	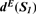	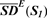	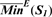	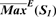	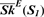
0.001	0.7±0.1	2.5±0.6	0.0±0.0	27.5±1.8	7.5±0.9
0.002	1.3±0.2	3.4±0.4	0.0±0.0	28.3±1.8	5.2±0.4
0.005	3.2±0.3	5.1±0.5	0.0±0.0	29.6±1.1	3.2±0.3
0.01	6.0±0.4	6.6±0.5	0.0±0.0	30.3±1.2	1.9±0.1
0.02	10.5±0.5	7.8±0.3	0.3±0.4	30.9±1.0	0.9±0.1
0.05	19.0±0.6	6.8±0.3	3.2±1.2	31.4±0.6	−0.4±0.1

The values of the statistical parameters correspond to the average over 50 independent runs for each value *μ*_1_ and were calculated as described in Methods. The standard deviation for each determination is shown after the sign ±.

**Table 2:** Statistical parameters describing the distribution of 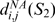 values (see Methods and Fig. 1).

*μ*_1_	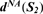	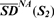	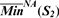	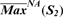	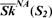
0.001	25.1±0.1	0.9±0.1	20.9±2.5	32.1±1.2	2.4±1.3
0.002	25.1±0.1	1.3±0.2	19.5±3.6	32.9±1.3	1.7±1.2
0.005	25.2±0.2	1.8±0.2	18.1±3.0	33.7±0.8	1.0±0.4
0.01	25.7±0.3	2.4±0.1	16.9±2.7	34.3±0.9	0.6±0.2
0.02	26.5±0.4	2.8±0.1	15.1±3.0	35.2±0.8	0.1±0.1
0.05	27.3±0.3	3.1±0.2	12.8±2.1	35.1±0.8	−0.6±0.1

The values of the statistical parameters were determined from the same 50 populations of Table 1, recalculating the distance values with respect to *S*_2_.

Equilibrated populations are optimized at a mutation rate *μ*_1_ towards the target structure *S*_1_ (Fig. 1). At the stationary state, the average distance of these populations to the target structure *S*_1_ depends on *μ*_1_ (see values for 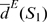 in Table 1). The higher *μ*_1_, the larger is the average distance to the target at the mutation-selection equilibrium. This result illustrates the dependence of the degree of adaptation on the mutation rate, as previously reported [42]. When the selective pressure is changed by choosing a new target structure (*S*_2_, see Fig. 1), populations optimized to reach target structure *S*_1_ are non-adapted when confronted to *S*_2_. As a rule, 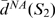 values in non-adapted populations (see Table 2) are much higher than 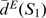 values in equilibrated populations (see Table 1), although the two populations differ much more at low than at high error rates. Actually, if the target structures *S*_1_ and *S*_2_ are at a large base pair distance of each other, optimized populations are typically farther from *S*_2_ than a population of randomly chosen sequences, especially for small *μ*_1_ (results not shown).

In addition to the the average distance, we have evaluated the average standard deviations [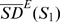 and 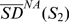] as a measure of the relative phenotypic diversity, the average minima [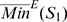 and 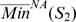] and maxima [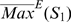 and 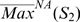], and the average skewness parameters [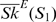 and 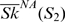], the latter standing for the bias of the distribution. The comparison between the corresponding values permits us to make several conclusions. 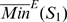 values shows that populations optimized at *μ*_1_ between 0.001 and 0.01 contain at least one molecule folding into the target, meaning that there is a finite fraction of molecules folding into *S*_1_ once the mutation-selection equilibrium has been reached. There is a fixation threshold *μ*_1_^F^ above which on average no molecule in the population folds into the target structure. Once the target structure changes, the range of 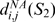 becomes much narrower, due to an increase in the minimum distance values. In general, in non-adapted populations, the larger the mutation rate *μ*_1_ the lower 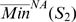, a behaviour clearly different from that observed in optimized populations, where 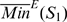 increases with the mutation rate *μ*_1_. The values of 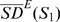 and 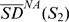 also increase with the mutation rate, although in optimized populations this increase is bound, decreasing above the fixation threshold, and suggesting that most molecules in populations evolved at high error rates have high 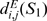 values, thus reducing population diversity. Finally, 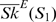 indicates that populations optimized at *μ*_1_ between 0.001 and 0.02 present a bias towards distance values above average. At *μ*_1_ = 0.05 this bias becomes negative, indicating the predominance of molecules with distance values below average. For non-adapted populations 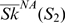 behaves in a way qualitatively similar to that of *E* populations.

### Adaptive dynamics of stationary-state populations

**Figure 2:**
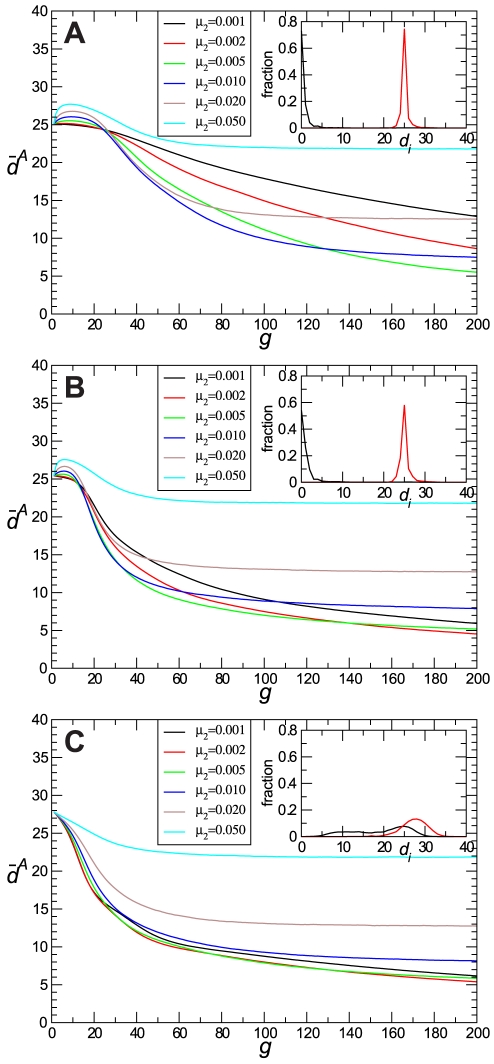
Temporal development of 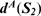. Results are shown for *μ*_1_ = 0.001 (A), 0.002 (B), and 0.05 (C), and for different values of *μ*_2_ (see legends). Each curve corresponds to an average over 1000 realizations. Insets: probability distributions of 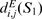 (black) and 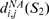 (red), representing the initial state (see also Tables 1 and 2).

We have analyzed the adaptive dynamics of the stationary-state populations described in Table 1 when the selective pressure is changed by choosing a new target structure (*S*_2_, see Fig. 1), as described. Populations optimized to reach target structure *S*_1_ are non-adapted when confronted to *S*_2_, and constitute the initial condition (*g* = 0) of a new adaptive process (see Table 2). With the aim of determining the optimal mutation rates that promote adaptation to *S*_2_, each population was allowed to replicate under a range of values of the new error rate (*μ*_2_) between 0.001 and 0.05. The variation of the value of 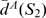 was evaluated through 200 generations. The results obtained for three representative populations differing in the value of *μ*_1_ at which they reached the previous stationary state are shown in Fig. 2. In the three cases considered we observe large differences in the adaptive dynamics depending on the value of *μ*_2_ used to adapt to the new selective pressure. For the largest value of *μ*_2_ considered (0.05), 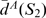 decreases only slightly as the number of generations increases, showing that too high error rates strongly hinder adaptation. For *μ*_2_ values lower than 0.05, there is a noticeable decrease in the average distance as time elapses. In general, the variation of 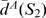 is faster at the beginning of the adaptive process, slowing down later. The initial decay occurs more rapidly for populations that reached the previous stationary state at moderate to high mutation rates than for those that were previously optimized at low mutation rates.

An interesting result observed in the evolution of the three populations represented in Fig. 2 is that, during the transient, the value of *μ*_2_ that promotes the maximum degree of adaptation to *S*_2_ (or the minimum value of 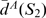 at a given time) depends on the number of generations elapsed. There are also differences among the three populations depending on the value of *μ*_1_ at which they were optimized towards *S*_1_. We can draw the general conclusion that the optimal mutation rate promoting maximal adaptation to a new selective pressure before reaching the new stationary state depends on the number of generations elapsed under the new conditions and on the previous mutation rate at which the population had evolved. In other words, the evolutionary history of populations has important consequences in their posterior adaptive capacity.

### Previous state of the population and optimal mutation rates at early stages of adaptation

**Figure 3:**
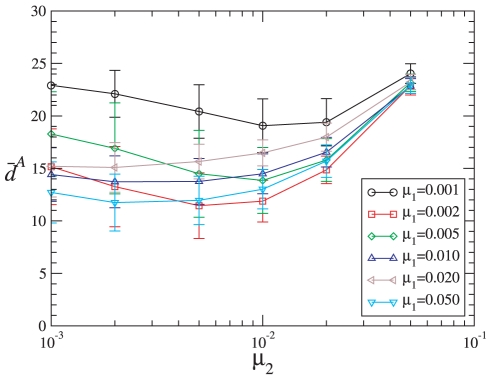
Average distance 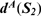 at *g* = 40 as a function of the mutation rate *μ*_2_ for different values of *μ*_1_. Each point corresponds to an average over 1000 realizations. The error bars yield the standard deviation 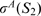 of the average distances obtained over all realizations, see Methods.

To explore the relationships between the value of *μ*_1_ at which populations adapted to *S*_1_ and the value of *μ*_2_ that promotes maximal adaptation to *S*_2_ after a short number of generations, we evaluated 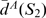 after 40 generations in populations that differed in *μ*_1_ and evolved at increasing values of *μ*_2_ (Fig. 3). We have chosen *g* = 40 to observe populations that are still far enough from equilibrium but have undergone a significant degree of adaptation. Other values of *g* meeting these requirements would be appropriate as well and do not qualitatively change our results.

In all populations the variation of 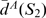 as a function of *μ*_2_ shows a non-monotonic behaviour, with important differences depending on the value of *μ*_1_ at which they reached the previous stationary state. For populations previously evolved at *μ*_1_≤0.005 there is a range of *μ*_2_ values across which 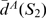 decreases significantly as the mutation rate increases (*p*≪0.001 for the comparisons of the difference of 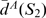 in populations optimized at *μ*_1_ = 0.001, 0.002, and 0.005 and evolving at *μ*_2_ = 0.001 and 0.01 for each value of *μ*_1_). There is a limit for these benefits, and at values of *μ*_2_ around 0.01, any additional increase of the mutation rate gives the same or higher value of 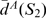. In contrast to these results, populations previously evolved at *μ*_1_≥0.01 (i. e. above the fixation threshold) keep an almost constant value of 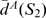 for low *μ*_2_ values (*p*>0.05 for the differences between 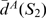 in populations optimized at *μ*_1_ = 0.01 and evolving at *μ*_2_ = 0.001 and 0.01, and in populations optimized at *μ*_1_ = 0.02 and 0.05 and evolving at *μ*_2_ = 0.001 and 0.005), while larger *μ*_2_ makes adaptation more difficult. As a consequence, it is not possible to establish a simple relationship between the value of *μ*_1_ at which populations evolved and the value of 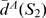 obtained under the new selective pressure, for any of the values of *μ*_2_ considered. This behaviour clearly differs from that observed in stationary state populations, where the higher the mutation rate, the higher also the value of 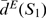 attained at equilibrium (Table 1) [42]. Our results clearly show that, for out-of-equilibrium populations, increases of the mutation rate may bring about adaptive advantages.

Another observation emerging from the results of Fig. 3 is that the influence of *μ*_1_ is maximal at low values of *μ*_2_. As this parameter increases, populations behave more similarly, and at *μ*_2_ = 0.1 all of them converge to approximately the same value of 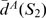 (results not shown). That is, the lower the value of the mutation rate *μ*_2_, the higher the influence of the previous state of the population.

The lowest values of 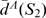 reached in all the cases after 40 generations, and the value of *μ*_2_ at which it is obtained, are shown in Table 3. We observe that the maximal degree of adaptation appears in two quite different situations. The first one corresponds to a population that reached the previous stationary state at *μ*_1_ = 0.002. When adapting to the new selective pressure, this population attains a very low 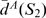 value in a short number of generations without altering the mutation rate (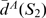 = 13.3 for *μ*_2_ = 0.002; see Fig. 3). The increase of *μ*_2_ to 0.005 results in a decrease of 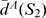 to the lowest value observed at *g* = 40 (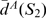 = 11.5). The second situation is represented by the population previously evolved at the highest mutation rate (*μ*_1_ = 0.05), having the lowest degree of adaptation to *S*_1_ at the stationary state (see values for 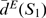 in Table 1). That population, when confronted with the new selective pressure *S*_2_, displays the almost lowest value of 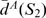 obtained in our simulations at *g* = 40 for all *μ*_2_<0.01. Interestingly, the population that performs worst for any value of *μ*_2_ is that with the lowest value of *μ*_1_ (Table 1), which permitted the highest degree of adaptation to *S*_1_. As indicated above, a small increase in the value of *μ*_1_ (from 0.001 to 0.002) yields a considerable increase in the adaptive capacity of this population (*p*≪0.001 for the difference of 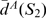 between populations evolved at the same *μ*_2_ and optimized at *μ*_1_ = 0.001 or 0.002), highlighting once more the significative non-proportional effects that small changes in the mutation rate might produce.

**Table 3:** Lowest values of 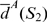 reached at *g* = 40 and values of *μ*_2_ at which they are obtained in populations previously optimized at the indicated values of *μ*_1_.

*μ*_1_	*μ*_2_	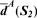
0.001	0.001	22.9
	0.01	19.6 (*p*≪0.001)
	0.02	19.4 (*p*≪0.001)
0.002	0.002	13.3
	0.005	11.5 (*p*≪0.001)
	0.01	11.9 (*p*≪0.001)
0.005	0.005	14.5
	0.01	13.9 (*p*≪0.001)
0.01	0.01	14.5
	0.002	13.7 (*p*≪0.001)
	0.005	13.8 (*p*≪0.001)
0.02	0.02	18.0
	0.001	15.2 (*p*≪0.001)
	0.002	15.1 (*p*≪0.001)
0.05	0.05	22.9
	0.002	11.8 (*p*≪0.001)
	0.005	12.0 (*p*≪0.001)

For all *μ*_1_ (left column) we show the lowest values of 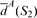 at *g* = 40 (right column), together with the values of *μ*_2_ at which they are attained (central column). We also display 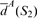 for *μ*_1_ = *μ*_2_ and the *p* value of the Student's *t*-test for the difference of 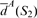 between the indicated population and the corresponding one evolving at *μ*_1_ = *μ*_2_.

## Discussion

The observation that mutation rates per nucleotide vary by orders of magnitude across species suggests that this character has not an optimal universal value [10], [47], [48]. Each species evolves under a mutation rate arising from many factors that are not universal. Among the most relevant we find the variability of the environment, the effect that mutations have on fitness, the metabolic costs of having more faithful replication machinery, the population size, and the replication rate [49]. Variations in any of these factors can modify the optimal value of the mutation rate, with the result that natural selection has to carry out a continuous fine tuning of this parameter and under the action of non-compatible trends may fail to find an optimal solution [11], [17].

In this paper we have focused on the study of the values of the mutation rates that promote maximal adaptation in a short number of generations when populations previously optimized at different error rates experience a single environmental shift. The model system we have used is constituted by ensembles of RNA molecules that evolve through mutation and selection towards a defined target structure. This system permits to establish direct correspondences between the genotype (the sequence of the RNA molecule), the phenotype (the structure into which it folds), the replicative ability (inversely related to the distance to the target), and the degree of adaptation of the whole population. Although in our simulations, mutation rates are imposed by the researcher, the relative amount of beneficial and deleterious mutations is not a fixed parameter, as it varies through the evolutionary process as a consequence of the variation in the degree of adaptation [9]. Consequently, the optimal mutation rate at the stationary state does not necessarely coincide with the optimal mutation rate before mutation-selection equilibrium is reached.

The populations considered in this paper have first evolved with different error rates (between 0.001 and 0.05) for a number of generations, large enough to reach mutation-selection equilibrium. Their degree of adaptation at equilibrium decreases with the mutation rate. However, when confronted with a new selective pressure, these populations can experience adaptive advantages if they vary their mutation rate. This means that the optimal mutation rate for an adapting population can be quite different from the optimal mutation rate under conditions that remain constant for a long time. In particular, for populations optimized at low mutation rates an increase of this parameter may be favourable, while for populations replicating under high mutation rates a decrease would be advantageous. These results can be partially explained as a consequence of the different dynamics of the adaptive process at different mutation rates, and by the influence of the composition of the population in its subsequent ability to adapt to new selective pressures. Adaptation is a complex phenomenon in which, in addition to the diversity generated *de novo*, the nature and distribution of existing mutants plays an important role. In our simulations, populations able to attain a high degree of adaptation to a new selective pressure in a short time were those previously optimized at moderate (0.002) to high (0.05) values of *μ*_1_ (Fig. 3 and Table 3). After changing the target structure, populations optimized at high mutation rates give rise to highly diverse, non-adapted populations, which contain in their mutant distributions molecules closer to *S*_2_ at *g* = 0 than populations optimized at lower error rates. These populations respond better when the mutation rate is decreased, thus enhancing the presence of structures close to *S*_2_. On the other hand, populations optimized at *μ*_1_ = 0.002 benefit from an increase in the mutation rate to rapidly adapt to the new target structure. At *g* = 0, these populations display lower diversity, and the molecules closest to *S*_2_ have typical distances larger than those in populations optimized at high error rates. Increases of the error rate promote the appearance of better adapted structures, the substrate of further optimization. These results provide good examples of the importance of pre-existent and newly generated diversity in the adaptive capacity of populations, and illustrate how the relative amount of each of them determines whether a population needs to increase or reduce the mutation rate to get rapid adaptation.

We have used an exponential fitness function, such that replication of molecules with any value of *d_i_* is possible. This is one reason why we do not observe extinction in any of the cases studied. The situation would be different should we work with a truncated landscape, for instance, where molecules folding too far from the target structure would not have the minimal functionality required for replication. More restrictive landscapes of this kind, together with a population that could vary its size, would yield extinction in cases of low *μ*_1_, especially. Still, the conditions for extinction in truncated landscapes would be alleviated in large enough populations, with a broader range of *d_i_* values and hence of diversity.

Our results can be discussed in the context of actual organisms that replicate using error rates that differ by orders of magnitude (from 10^−8^–10^−9^ in DNA organisms to 10^−4^–10^−6^ in RNA viruses). Although the error rates for the RNA molecules used in this study are apparently much higher than the error rates of actual organisms, they become much more similar if the values are expressed per genome, or replicating molecule. Taking genome size into account, our RNA molecules replicate with error rates between 0.05 and 2.5 errors per molecule and generation, a value quite similar to the error rates found in Nature, which range between roughly 0.003 errors per genome and generation in DNA based microbes to 1–5 in RNA viruses [10]. These values can be further increased by mutagenic agents.

Populations optimized at low mutation rates present a high degree of adaptation and a low phenotypic diversity (see Table 1). After a limited number of generations, under a new selective pressure, the highest degree of adaptation reached by these populations takes place when they increase the mutation rate to values of 0.01 or 0.02 (Fig. 3 and Table 3). These populations behave similarly to DNA organisms that maintain a certain degree of constancy in the intracellular medium, which allows a reduced mutation rate for replication. However, under conditions of environmental stress, a more convenient evolutionary strategy for these organisms would be to increase the mutation rate to get rapid adaptation [14]–[16]. This strategy is particularly important when the new selective pressure is strong enough to extinguish the population in case it is not able to adapt in a short time span. A well known example is the selection of hypermutator strains when bacterial populations are infected with phages or exposed to antibiotics [43], [44], [50]. There is however a limit for the beneficial effects derived from the increase of the error rate. In our system, when the mutation rates are increased above *μ*_2_∼0.01, the degree of adaptation reached diminishes (see Fig. 3), a result that suggests that there must be also a limit for the increase of the mutation rate that can be attained by a hypermutator strain. In our case, this limit is set by the mutation rate above which fixation of the optimal phenotype becomes impossible.

There are important differences in the behaviour of populations evolving at low error rates. In our system, the population that performs worst under a new selective pressure at any of the values of *μ*_2_ assayed is that one previously optimized at the lowest mutation rate considered (*μ*_1_ = 0.001; see Fig. 3). This population reached the highest degree of adaptation at the stationary state (see Table 1) and resembles specialist organisms that perform optimally under a narrow range of very well established conditions, but have difficulties to adapt when these conditions are modified [51]. Populations optimized at a slightly higher error rate (*μ*_1_ = 0.002) experience a substantial increase in their adaptive ability (Fig. 3; Table 3). This population could represent less specialized organisms, not so well adapted to a concrete selective pressure, but able to perform optimally in a wider diversity of environments [52] due to their higher degree of genotypic and phenotypic diversity. These results also suggest that the transition from a rigid population, with little ability to respond to environmental changes, to a more flexible population, able to adapt rapidly, may occur through small increases of the mutation rate that produce concomitant increases in the pre-existent diversity.

Populations optimized at high mutation rates (*μ*_1_ = 0.01) can adapt rapidly to a new selective pressure keeping this parameter constant. These cases resemble in some aspects RNA virus populations, which, together with viroids, replicate at the highest error rates found in Nature and that are able to adapt rapidly to changing environments without significantly altering the error rate. The disadvantages experienced by these populations when the error rate is further increased [30], [53] agree with the fact that only mild mutator mutants have been isolated in the case of RNA viruses [27], [28]. In normal conditions, RNA viruses also do not select mutants with lower than standard mutation rate. It is likely that the associated reduction in the genotypic and phenotypic diversity strongly challenges their ability to undergo adaptation [35], [36].

As could have been expected, populations optimized at values of *μ*_1_≥0.02 (above the fixation threshold) can dramatically increase their adaptive potential if they reduce the mutation rate. These populations sustain a high degree of diversity, with molecules already close to other possible new target structures. If the mutation rate is kept high, molecules with low distance values are lost due to the strength of mutation. However, when the mutation rate is reduced, these molecules can become fixed, permitting in this way the optimization of the whole population. The equivalent in Nature of these populations could be RNA viruses replicating at higher than standard mutation rates, a condition that has been explored experimentally by exposing RNA virus populations to mutagens. RNA viruses can escape the negative consequences of the increase of the mutation rate by selecting anti-mutator mutants [34]. The consequences of the selection of an anti-mutator mutant in a population previously mutagenized have not been explored, but they could be quite negative from the viewpoint of the host if, as predicted by our model, they are associated to a short term increase in the adaptive ability. The treatment of RNA virus infections with mutagens is being investigated as a new therapeutic approach known as lethal mutagenesis [53]. One of the main criticisms to this alternative therapy is that the increase of diversity caused by the mutagen could induce a parallel increase in the adaptive ability of the virus [54]. Our results suggest that if the mutagen is withdrawn before infection clearance, or if anti-mutator mutants emerge, the resulting populations could adapt more easily to new selective pressures. A great care should be taken when manipulating the error rate of pathogenic organisms. The associated variations in fitness and adaptive capacity could result in the generation of strains better suited to resist new treatments or the action of the immune system of the host. Therefore, a continuous research in this field combining both experimental and computational approaches is highly promising.
